# Steady electrocorticogram characteristics predict specific stress-induced behavioral phenotypes

**DOI:** 10.3389/fnins.2023.1047848

**Published:** 2023-04-11

**Authors:** Laura Desnouveaux, Betty Poly, Mathilde Edmond, Cathy Aphezberro, David Coulon, Francis Boutet, Christine Le Coz, Francisca Fargeau, Cyril Linard, Pierre Caillol, Anaïs M. Duffaud, Aurélie Servonnet, Ouamar Ferhani, Marion Trousselard, Nicolas Taudon, Frédéric Canini, Damien Claverie

**Affiliations:** ^1^Unité de Développements Analytiques et Bioanalyse, Département Plateformes et Recherche Technologique, Institut de Recherche Biomédicale des Armées (IRBA), Brétigny-sur-Orge, France; ^2^Unité de Neurophysiologie du Stress, Département Neurosciences & Contraintes Opérationnelles, Institut de Recherche Biomédicale des Armées (IRBA), Brétigny-sur-Orge, France; ^3^Département Innovation Numérique et Intelligence Artificielle, Institut de Recherche Biomédicale des Armées (IRBA), Brétigny-sur-Orge, France; ^4^Unité Analyses Biologiques, Département Plateformes et Recherche Technologique, Institut de Recherche Biomédicale des Armées (IRBA), Brétigny-sur-Orge, France; ^5^APEMAC, EA 4360, Université de Lorraine, Nancy, France; ^6^Ecole du Val de Grâce, Paris, France; ^7^Réseau ABC des Psychotraumas, Montpellier, France

**Keywords:** EEG, fear, multisensorial stress, β2, theta, vulnerability

## Abstract

**Introduction:**

Depending on the individual, exposure to an intense stressor may, or may not, lead to a stress-induced pathology. Predicting the physiopathological evolution in an individual is therefore an important challenge, at least for prevention. In this context, we developed an ethological model of simulated predator exposure in rats: we call this the multisensorial stress model (MSS). We hypothesized that: (i) MSS exposure can induce stress-induced phenotypes, and (ii) an electrocorticogram (ECoG) recorded before stress exposure can predict phenotypes observed after stress.

**Methods:**

Forty-five Sprague Dawley rats were equipped with ECoG telemetry and divided into two groups. The Stress group (*n* = 23) was exposed to an MSS that combined synthetic fox feces odor deposited on filter paper, synthetic blood odor, and 22 kHz rodent distress calls; the Sham group (*n* = 22) was not exposed to any sensorial stimulus. Fifteen days after initial exposure, the two groups were re-exposed to a context that included a filter paper soaked with water as a traumatic object (TO) reminder. During this re-exposure, freezing behavior and avoidance of the filter paper were measured.

**Results:**

Three behaviors were observed in the Stress group: 39% developed a fear memory phenotype (freezing, avoidance, and hyperreactivity); 26% developed avoidance and anhedonia; and 35% made a full recovery. We also identified pre-stress ECoG biomarkers that accurately predicted cluster membership. Decreased chronic 24 h frontal Low θ relative power was associated with resilience; increased frontal Low θ relative power was associated with fear memory; and decreased parietal β2 frequency was associated with the avoidant-anhedonic phenotype.

**Discussion:**

These predictive biomarkers open the way to preventive medicine for stress-induced diseases.

## 1. Introduction

In humans, intense stressor exposition produces several phenotypes. Exposure to an intense stressor may result in an acute stress disorder that disappears totally, partially, or not at all over time. It can also lead to an absence of fear memory or leave psychological sequelae that range from poor fear memory to post-traumatic stress disorder (PTSD; Yehuda et al., 2015). Furthermore, these effects may vary over time (Muresanu et al., 2022). Exposure to an intense stressor also opens the way to Major Depressive Disorder (MDD), panic disorder, substance use disorders, or burnout (Berenz et al., 2019; Restauri and Sheridan, 2020; Spencer et al., 2022). The question, therefore, is: why do such differences exist among individuals? Thus, this study aimed to understand the variability of outcomes after exposure to a standardized stressor as a function of risk factors.

Both contextual and intrinsic factors can be consistent with a negative outcome. *Contextual* factors refer to the intensity and proximity of the stressor, and the ability to escape from it (Blanchard and Blanchard, 1989; Fanselow et al., 2019). These factors are observed in both rodents and humans. In the latter case, for example, being raped is far more frequently followed by PTSD than being a witness to a rape (Yehuda et al., 2015). *Intrinsic* factors relate to the individual and include the intensity of the initial response and a potential preexisting vulnerability. In humans, an intense response to a stressor involves the activation of the sympathetic nervous system, the mobilization of the hypothalamic-pituitary-adrenal (HPA) axis, along with panic, and dissociative clinical signs (Speer et al., 2019). In rodents, it is mainly characterized by freezing behavior (Verbitsky et al., 2020).

Vulnerability refers to an increased risk of transition to a pejorative outcome after stress exposure (Kraaijenvanger et al., 2020). Vulnerability is not necessarily specific to a particular disease. In humans, neuroticism is a risk factor for PTSD (Yin et al., 2019) and MDD (Ka et al., 2021), while MDD and PTSD are comorbid in ~50% of cases of PTSD (Radell et al., 2020). Vulnerability can often be traced to the individual's genetics. Genetic alterations, linked to an enhanced risk of PTSD or MDD, affect systems involved in stress reactions, notably the sympathetic and HPA axes, and Brain-Derived Neurotrophic Factor (BDNF; Maul et al., 2020). However, very few studies have examined brain function before exposure to a stressor. Amongst these studies, parietal electrocorticogram (ECoG) β2 frequency predicted vulnerability to an MDD phenotype in a social defeat model (Claverie et al., 2016). Although the biological meaning remains elusive, this difference was observed at baseline and did not change throughout the experimental time course. In contrast to vulnerability, resilience is defined as the ability to recover from a challenging stressor (Deems and Leuner, 2020).

Studies of both vulnerability and resilience raise methodological concerns. Vulnerability is usually studied *a posteriori* to the challenge (Lorsch et al., 2021). This retrospective approach seeks to isolate correlations between post-stress exposure phenotypes and pre-stress (baseline) measures. It also requires a complementary, prospective approach that evaluates the outcome of a given population based on a potential genetic or functional biological mechanism. Such studies are difficult to carry out in a human sample due to (i) the low frequency of the pejorative outcome, requiring a huge population (Kilpatrick et al., 2003; Kline et al., 2021) and (ii) the high degree of variability in the potentially traumatic event, which itself leads to an even more variable degree of perceived stress. Earlier work suggests that, while 89% of the American population will be exposed to at least one potentially traumatic event in their lifetime (Kilpatrick et al., 2003; Berenz et al., 2019), only 6% will develop PTSD (Koenen et al., 2017). Therefore, animal models must be used to study vulnerability to stress-induced pathologies, notably PTSD and MDD. In this context, a stress model, such as social defeat, is generally used to model fear memory and depression phenotypes.

The percentage of stress-induced pathologies depends on the stressor's characteristics. These stressor characteristics are intensity and proximity, which indicates the possibility that the animal can escape (Blanchard et al., 1990; Fanselow et al., 2019). In practice, animal models can induce ~50% of the transition from the baseline to stress-induced pathologies, and their reproducibility is a cornerstone that underpins comparisons of results across laboratories (Keenan et al., 2018). However, reproducibility reflects a compromise with ecological validity. In the case of social defeat, which is the most ecological model, stressor exposure intensity depends mainly on how aggressive the resident animal is. It is tempting to improve reproducibility by increasing stressor aggressiveness. For instance, models based on footshock show that the intensity of the voltage contributes 20–100% to fear memory (Davis and Astrachan, 1978; Bush et al., 2007). High-intensity stimuli lose their specificity: the response becomes nociceptive and interacts with stress pathways through pain and fear (Han et al., 2015). The latter observation is also true for trimethylthiazoline (TMT). This synthetic compound is found in fox feces, which may induce a noxious reaction besides predator fear (Fendt and Endres, 2008). In that case, ecological validity may be irrelevant and fade out.

Although developing a realistic, reproducible model is clearly a challenge, it could be useful in PTSD vulnerability studies. Such a model should include a variable, acute response in the form of a stress reaction that involves both adrenergic and HPA axes (Horii et al., 2010; Tyler et al., 2020); a behavioral activity that reflects the method used to cope with the challenge; and a long-term analysis of fear memory and depression. Therefore, we built a new realistic ethological model of fear confrontation based on a multisensorial challenge. It simulated a fox attack by exposing rats to a scenario that combined synthetic fox odors (TMT), synthetic blood odors (TED: Trans-4,5-epoxy-2(E)-Decenal), and 22 kHz rodent distress vocalizations, during a non-escapable 10-min exposure. The long-term consequences of stressor exposure were evaluated using re-exposure to the context.

The aims of the present study were to evaluate (i) the capacity of the MSS to induce stress-related pathologies and (ii) whether baseline ECoG differences can predict MSS outcomes.

## 2. Materials and methods

### 2.1. Animals

A total of 46 male Sprague-Dawley rats (Janvier Laboratoire, France) weighing 200 g (8 weeks old) at the beginning of the experiment were included. The housing environment was controlled: light/dark cycle (12 h−12 h dark-light cycle with lights on at 07:00 a.m.), ambient temperature (21 ± 1°C), and relative humidity (50 ± 10%). Rats had *ad libitum* access to food and water. Since the procedures involved animals, animal care protocols complied with institutional guidelines that themselves comply with national and international laws and policies, and the study received authorization from the Institutional Ethics Committee (no 24.2017).

### 2.2. Experimental design

**Figure 1:**
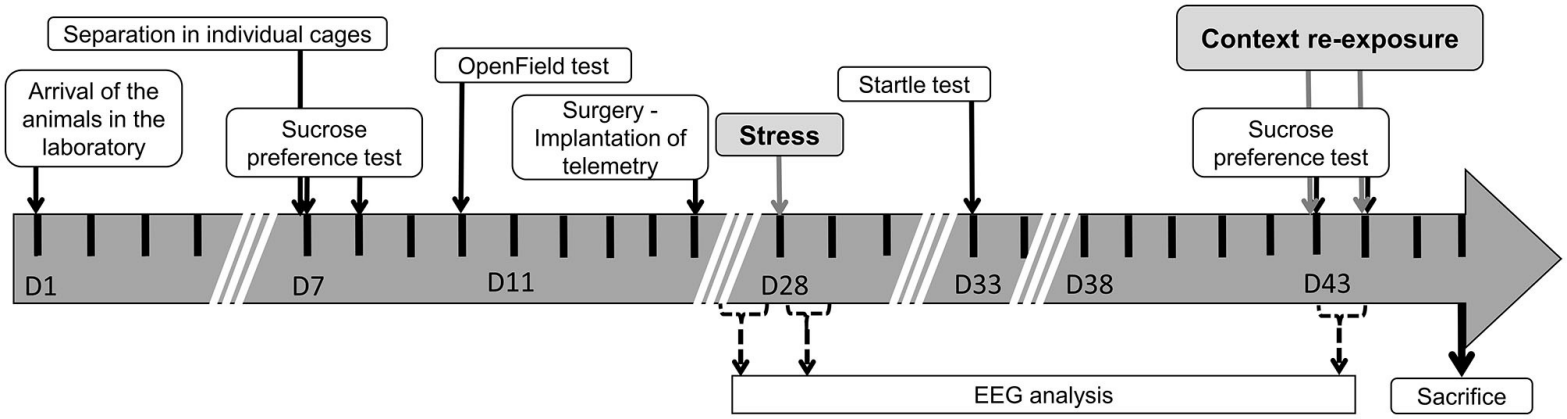
Experimental time course.

All rats followed the same experimental time course (Figure 1). On arrival, animals were housed in groups of 4–5 for 6 days (D1–D6) to become accustomed to laboratory conditions. They were then transferred to individual cages (length, 45 cm; width, 25 cm; height, 17 cm) but kept in contact with social cues (sound, odors, etc.). A sucrose preference test (SPT) was performed on D7 and D8 (SPT1). An open-field test was run on D10. At D15, rats were surgically equipped with a telemetric recorder [TL11M2-F20-EET, DataScience International (DSI), Minneapolis, USA]. One animal died after surgery. They were then allowed a 13-day period to recover. At the end of this period (D28), animals were randomly assigned to one of two experimental groups: stressed rats (Stress, *n* = 23) were exposed to the MSS procedure, while sham rats (Sham, *n* = 22) were exposed to a sham MSS procedure. Both sham and stressed animals were housed in the same conditions but in different rooms to avoid communication between experimental groups. Similarly, both groups were separately handled every day throughout the investigation. The animals underwent an acoustic startle test (SR-LAB^TM^ Startle Response System, SD Instruments, San Diego) 5 days after MSS exposure (D33) and a second SPT 10 days later (SPT2; D43–D44). Finally, 15 and 16 days after MSS exposure (D43–D44), animals were re-exposed two times to the MSS context with no aversive stimuli (R1, R2, respectively). Animals were killed by vigil decapitation 2 days after the last test (D46).

### 2.3. Stress conditioning

#### 2.3.1. MSS and sham-MSS procedures

The MSS apparatus consisted of a Plexiglas box measuring 30 × 30 × 50 cm (l × w × h). Two opposite sides were transparent, while the others were opaque. The box was equipped with a lid, which was itself equipped with a camera (Logitech C920 1,080 p), two loudspeakers (Visaton SC 4.7 ND) broadcasting the 22 kHz rodent vocalization, and two ultrasonic fragrance diffusers (mist makers running at 113 kHz) measuring 20 mm. All were linked to a matrix that was connected to a computer and managed by software developed by the Digital Innovation and Artificial Intelligence Department of the French Military Biomedical Research Institute.

The 22 kHz vocalizations corresponded to calls emitted by rats in aversive situations (Fendt et al., 2018). Validated sounds were generously provided by loudspeakers (Fendt et al., 2018) and were broadcast throughout the procedure. Blood odor (TED: trans-4,5-Epoxy-2(E)-decenal) is known to induce avoidance in rats (Stevens and Saplikoski, 1973), and it was diffused by ultrasonic mist makers at 3, 6, and 9 min during the MSS exposure (supplied by CaymanChemical; Ref: 10004257; Lot: 0528221-10). TMT was used at 30 μL (SRQ Bio, Sarasota, FL, United States Ref: 300000368; Lot: 13267 and 14160). It was dropped on a filter paper (2 × 2 cm), which was placed in a plastic cup that was always located in the same corner of the box. Since TMT mimics the predator odor, this filter paper was interpreted as a traumatic object (TO) for the rat.

The MSS consisted of a 10-min exposure to the 22 kHz sound, the three blood odor diffusions, and continuous TMT exposure, allowing exploration or avoidance of the TO. The sham-MSS context was similar. However, in this case, no sounds were diffused, and odor solutions were replaced by water, either in the diffusor or on the 2 × 2 cm filter paper, which was similarly placed (i.e., in a plastic cup located in the same corner of the box). In both cases, the box was cleaned with alcohol and then water before and after each animal was placed. Animals were filmed throughout the initial MSS exposure and the subsequent re-exposures. Stressed and sham rats were tested in two different rooms.

#### 2.3.2. Re-exposure to the MSS/sham-MSS context

Re-exposure to the MSS/sham-MSS context was carried out on two consecutive days (R1, R2). Animals were placed for 10 min in a new but similar apparatus (a similar box, with filter paper in a plastic cup placed at the left side of the apparatus). The sham exposure procedure was also repeated (no sound, water in the diffuser, 30 μL of water on the filter paper) and animals were filmed throughout the test. Stressed and sham rats were tested in two different rooms.

#### 2.3.3. Video data analysis

Videos recorded during MSS, sham-MSS, and R1/R2 re-exposures were analyzed using EthoVision XT15 software (Noldus Information Technology), a validated software for animal behavior analysis (Noldus et al., 2001).

Fear and fear memory were evaluated during MSS/sham-MSS and R1/R2, respectively. The dependent variables were the duration of freezing (in percentage), and the duration and number of interactions with the TO.

Freezing was defined as the absence of movement, other than respiratory movement, for more than 3 s (Schwendt et al., 2018). In all cases, it was assessed using the automatic “Activity state” function included in the Ethovision XT15 software package. This function optimizes the detection of the animal's movements under the lighting and reflection conditions specific to the enclosure. Freezing parameters (Averaging interval: 40, Inactive below: 0.60%, Exclude instances shorter than: 3.00 s) were chosen to adjust the results of the automatic assessment to those of the expert assessment (mean difference: 2%, standard error of the mean: 2%). Manual scoring was carried out on 10-min long videos. Consistent with an earlier study, the dependent variable was defined as the duration of freezing (percentage of the total test duration) during exposure and re-exposure (Schwendt et al., 2018).

Behavioral interactions with the filter paper included: sniffing, directed toward the object; moving the object; and touching the object with the nose or front leg. The duration and number of interactions were manually assessed.

### 2.4. Behavioral evaluation

#### 2.4.1. The sucrose preference test

This test assesses anhedonia, which is characterized by a decreased sucrose preference. Anhedonia was identified when sucrose preference fell below 65% (Scheggi et al., 2018). Two of the animal's usual bottles were used. The first was filled with water and sucrose [D(+)-Saccharose VWR; Ref: 27480.360; Lot: 19F274102] at a concentration of 1.5%, and the second was filled with plain water. Rats had *ad libitum* access to both for 48 h. The location of sweet and plain water bottles was swapped every day to avoid place preference. Intakes were measured daily at the same time. Average sucrose intake was expressed as the percentage of total liquid consumption [sucrose intake/ (sweet + plain water consumption) * 100] over two consecutive days of the experiment. When measures between 2 consecutive days differed by more than 50%, they were considered as place preference and excluded. The test was carried out two times, at Baseline (SPT1) and after R1 and R2 re-exposures (SPT2).

#### 2.4.2. The open-field test

The open-field test evaluates anxiety (Prut and Belzung, 2003). The apparatus consisted of a white, square arena (1 × 1 m) surrounded by a 40 cm wall. The arena was illuminated with a white 30 lux light placed in the center of the test area. The apparatus was placed in a dedicated room. A camera above the arena recorded the animal's movements. This ethological analysis was carried out using Ethovision XT15 software. The animal was placed in the center of the arena and left to move freely for 10 min. Then, it was removed and placed back in its cage. The arena was cleaned with 70% ethanol, then water, before each testing session. The dependent variable was measured as the time spent in the center of the arena as this has been related to the anxiety level of the animal (Prut and Belzung, 2003).

#### 2.4.3. The acoustic startle test

The acoustic startle test evaluates the behavioral reactivity of animals to an intense auditory stimulus (Valsamis and Schmid, 2011). The apparatus consisted of a hermetically-sealed enclosure with acoustic insulation (SR-LAB, San Diego Instruments, USA). The enclosure was equipped with force sensors to record the animal's movements, and a loudspeaker was placed above the animal to broadcast sound stimuli at various intensities.

The full test session lasted 15 min. This was broken down into a 5-min period to accustom the animal to the enclosure, and a block of 30 stimuli at 120 dB. Each stimulus lasted 40 ms, and the interstimulus interval ranged from 10 to 30 s. Reactivity was evaluated using SR-LAB software. The dependent variable was the amplitude of movements (recorded by force sensors) during the stimulus broadcast. Amplitude values were normalized to baseline (the last 10 amplitudes were normalized to the first two), as this normalization provided an insight into the animal's habituation to the stimulus repetition (Valsamis and Schmid, 2011; Schwendt et al., 2018).

### 2.5. Telemetric assessment of the electrocorticogram (ECoG)

#### 2.5.1. Surgery

Rats were implanted with a TL11M2-F20-EET telemetric transmitter (Data Sciences International, Minneapolis, USA) allowing spontaneous locomotion (SLA) and electrocorticogram (ECoG) assessments.

The transmitter was inserted in each rat's abdominal cavity under deep anesthesia (buprenorphine 0.05 mg/ kg, isoflurane 4% at induction, and then 2%) through a sagittal abdominal incision. Muscles and the skin were then stitched up separately. The first electrode pair was comprised of a reference electrode secured above the cerebellum, and a recording electrode was secured on the parietal cortex above the CA1 hippocampus (−4.0 mm posterior, +2.0 mm lateral). The second pair consisted of a reference electrode secured above the cerebellum, and a recording electrode secured onto the frontal cortex above M2 (+2.6 mm posterior, +1.5 mm lateral). Electrodes were placed above the two regions (prefrontal cortex and hippocampus) involved in stress reaction modulation. These placements followed previous study (Claverie et al., 2016). Brain electrodes were maintained in the skull using stainless steel screws and dental cement (UNIFAST™ Trad, GC America). Rats received antibiotic (benzylpenicilline, 22000 UI, s.c.) and anti-inflammatory (carprofen, 4 mg/kg/d, s.c.) medication for 3 consecutive days following surgery and were allowed 13 days to recover.

#### 2.5.2. Telemetric assessments

RPC-1 receiver plates and the Data Exchange matrix were connected to a computer running ART Gold software (version 3.1, Data Sciences International, Saint-Paul, MN, USA). The ECoG and SLA were recorded in all animals. Due to a battery failure, signals from one rat were not recorded. Then, 24-h recordings were split into 5-s epochs to be able to analyze three concomitant telemetric variables. SLA was sampled at 1 Hz, corresponding to a variation in the signal strength of the telemetric recording (DSI device specifications), which was assumed to be related to the speed of movement of the animal (Studholme et al., 2013; Vivanco et al., 2013). Locomotion was calculated for each epoch in the DSI locomotor unit (AU). ECoG signals were recorded at a sampling rate of 1,000 Hz and then downsampled to 500 Hz. SLA and ECoG analyses were carried out on artifact-free 5-s epochs. Following the three reference methods (Tong and Thakor, 2009), epochs were considered as artifacts when at least one value was missing, when the absolute variation of the signal slope was >0.075 μV/ms, or when the absolute value of the signal was >5 standard deviations of the mean. An ECoG power spectral analysis was then run for each non-artifacted epoch using a Fast Fourier Transform with Welch estimation (500 points, hamming window, and 50% overlap) using MATLAB software (MathWorks, version r2019a). The following bands were considered: δ: 1.5–4.0 Hz, Low θ: 4.0–6.5 Hz, High θ: 6.5–9.5 Hz, α: 9.5–12.0 Hz, β1: 13–18 Hz, β2: 18–25 Hz, and Slow γ: 25–48 Hz as, according to the manufacturer, the telemetric bandwidth was 1–50 Hz. The absolute power of each band was calculated from the ECoG spectrum as the area under the curve. The power of each band was expressed as a ratio relative to the total power spectrum in each epoch. When present, the maximal peak power frequency was identified using a MATLAB function (*findpeaks*) in each band. This frequency was termed the “main peak frequency,” as previously reported (Claverie et al., 2016).

#### 2.5.3. Definition of active waking

Active waking was defined as validated in earlier work (Claverie et al., 2016). Briefly, 5-s epochs, in which the SLA value was greater or equal to 1 AU (Arbitrary Unit, defined by the manufacturer), were considered as active waking. This threshold has been demonstrated to have both good specificity (99.3%) and predictive power (98.1%) in an earlier study (Claverie et al., 2016).

#### 2.5.4. Spontaneous life in the home cage

Three, 24 h recordings were carried out at baseline (D26–D27), post-stress (D28–D29), and post-R1 re-exposure (D43–D44). SLA was calculated as the mean of the 5-s epoch of active waking for each animal.

### 2.6. Biological analysis

Trunk blood was collected after decapitation. The collection tube was left for 30 min at 21°C to allow coagulation. Then, tubes were centrifuged at 3,500 *g* for 7 min at 4°C. The supernatant was collected and stocked at −80°C until analysis.

#### 2.6.1. Serum BDNF

Serum BDNF (sBDNF) was assessed with an ELISA kit (Mature BDNF Rapid ELISA Kit, BEK-2211, Biosensis, Australia). The analysis was performed according to the manufacturer's instructions.

#### 2.6.2. Serum corticosterone level

Serum corticosterone levels were determined with a UHPLC (LC40, Shimadzu) coupled to a mass spectrometer (4000 QTrap, Sciex) after solid phase extraction using an HLB 30 mg/ 1 cc cartridge (Waters). Chromatographic separation used an Acquity BEH C18 column (50 × 2.1 mm, Waters), with 1.7 μm particle size at 40°C. Quantitation was carried out with corticosterone-d8, the labeled internal standard.

### 2.7. Statistical analysis

#### 2.7.1. Group clustering

In the Stress group, the fear memory was evaluated with respect to subgroups of behaviors, identified using TO interactions during R2 (i.e., the last, persistent phenotype). The following variables were considered: (i) the freezing percentage, (ii) the number, and (iii) the duration of interactions with the TO.

The optimal number of subgroups was determined using three algorithms (Silhouette, Gap, and Davies– Bouldin) adapted for the Gaussian mixture model clustering method, using MATLAB software (Supplementary Figure 1). Criteria were calculated for 1–6 cluster solutions (using default criteria). Due to mathematical constraints, the Silhouette and Davies–Bouldin criterion values cannot be calculated when the optimal number of clusters is 1.

The Silhouette criterion measures how similar a point is to other points in the same cluster, compared to points in other clusters. The higher the value, the better the solution. High values were observed for three cluster solutions (Supplementary Figure 1A). The Davies–Bouldin criterion is the ratio of intra- and inter-cluster distances. Here, the optimal number of clusters is the smallest value, and the lowest values were observed for three cluster solutions (Supplementary Figure 1B). The gap criterion measures how different total intra-cluster variation is with respect to a random, uniform distribution. Here, the optimal number of clusters is the smallest value of *k*, for which the statistic is within one standard deviation of *k*+1. Here again, *k* was optimal for three clusters (Supplementary Figure 1C). Hence, all algorithms determined that three, Gaussian-repartitioned clusters were optimal; these were called C1 (Fear), C2 (Avoidance), and C3 (Neotic; cf. Results).

#### 2.7.2. Predictivity of behavioral clustering

As the cluster analysis was only run on the overall Stress group, predictive variables were only analyzed in Stress subgroups. Predictors of future behavioral subgroups were chosen from ECoG telemetric data obtained during the 24-h baseline recordings. The predictive performance of each variable was evaluated using ROC curves. All variables were tested for each subgroup, but only those with an area under the curve (AUC) of > 0.8 were considered. Each subgroup could be predicted by at least one ECoG biomarker, with a good ability (around 80% of accuracy).

#### 2.7.3. General statistical analyses

STATISTICA software was used for all statistical analyses (version 7.1, StatSoft-France, Maisons-Alfort, France). Analyses were divided into three steps: (i) inter-group comparisons of the Stress and Sham groups; (ii) intra-group comparisons of the Stress subgroups; and (iii) inter-group comparisons of the Stress subgroups and the Sham group.

Inter-group differences were assessed using analysis of variance (ANOVA). Time courses were analyzed using a repeated measures ANOVA of *Time* (Baseline, Post-Stress, and Post-Re-exposure), *Group* [C1 (Fear), C2 (Avoidance), C3 (Neotic)], and the *Group* × *Time* interaction. When the ANOVA revealed a significant effect, *post-hoc* Bonferroni tests were run for all pairs. Comparisons between each Stress subgroup and the Sham group were performed using an ANOVA, followed, when necessary, by a bilateral *post-hoc* Dunnett test using the Sham group as a reference. The significance level was set at a *p*-value of < 0.05, and a trend was identified when a *p*-value was < 0.10. Data were presented as mean ± standard error of the mean (SEM).

## 3. Results

### 3.1. Analysis of stress effects

#### 3.1.1. MSS/sham-MSS and context exposure

**Figure 2:**
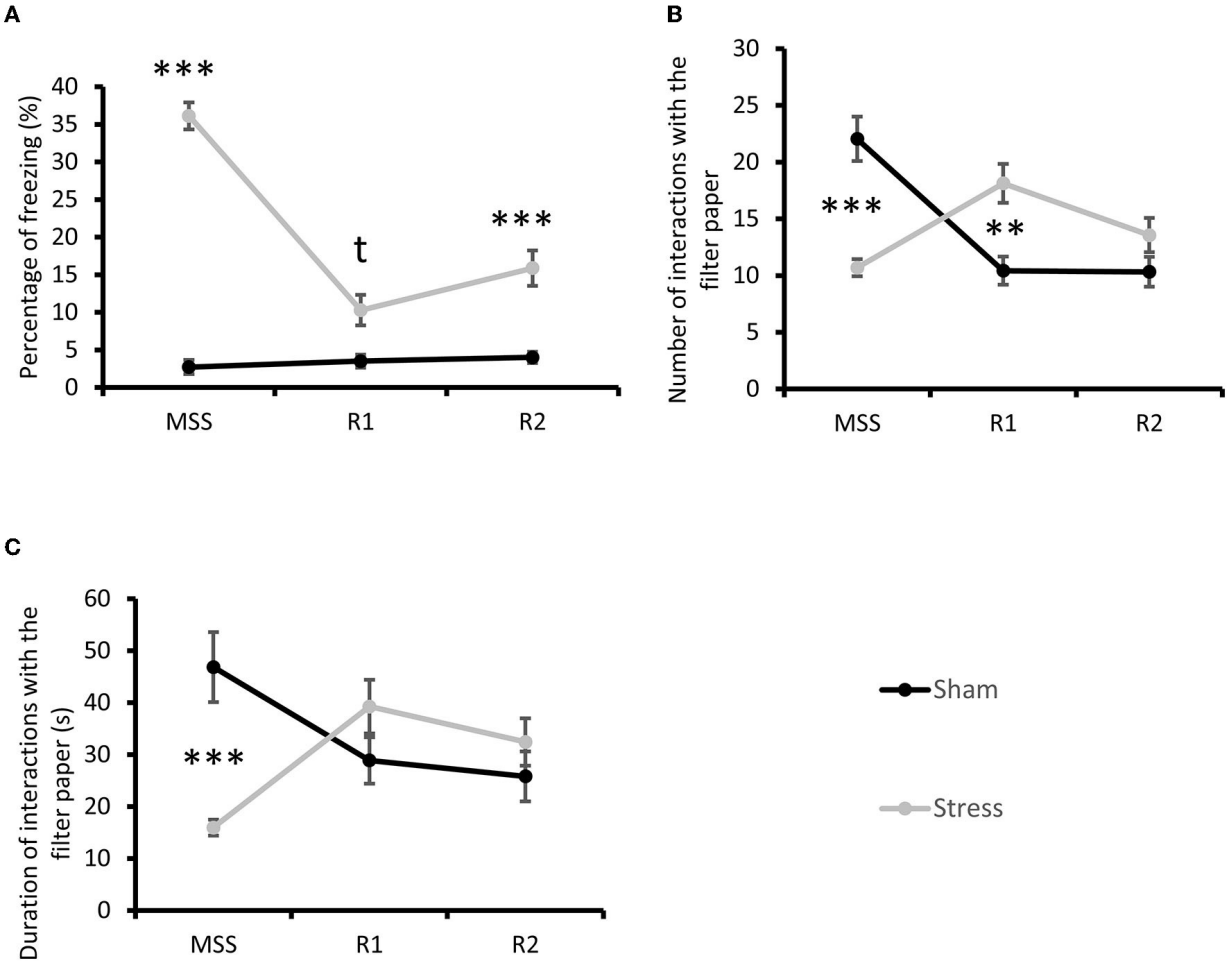
Multisensorial stress context exposure. **(A)** Percentage of freezing during the 10-min MSS, sham-MSS, and re-exposures. *Post-hoc* Bonferroni test ^*t*^*p* < 0.10, ***p < 0.001. **(B)** Number of interactions with TO during the 10-min MSS, sham-MSS, and re-exposures. *Post-hoc* Bonferroni test **p < 0.01, ***p < 0.001. **(C)** Duration (in sec) of interaction with TO during the 10-min MSS, sham-MSS, and re-exposures. *Post-hoc* Bonferroni test ***p < 0.001. Results are expressed as mean ± SEM.

The repeated measures ANOVA showed that: (i) the freezing percentage was significantly higher in the Stress group than the Sham group during the initial MSS/sham-MSS exposure [repeated measures ANOVA: Group × Time effect *F*_(2, 86)_ = 63.32, *p* < 0.001, partial Eta-squared = 0.60, *post-hoc* Bonferroni test *p* < 0.001; Figure 2A]; (ii) the number of TO interactions was lower in the Stress group than in the Sham group [repeated measures ANOVA: Group × Time effect *F*_(2, 84)_ = 30.49, partial Eta-squared = 0.42, *p* < 0.001, *post-hoc* Bonferroni test *p* < 0.001; Figure 2B]; and (iii) the cumulative duration of TO interaction was shorter in the Stress group than in Sham group [repeated measures ANOVA: Group × Time effect *F*_(2, 84)_ = 13.52, *p* < 0.001, partial Eta-squared = 0.24, *post-hoc* Bonferroni test *p* < 0.001; Figure 2C].

On re-exposure, the freezing percentage was higher in the Stress group than the Sham group at both R1 (*post-hoc* Bonferroni test *p* = 0.054) and R2 (*post-hoc* Bonferroni test *p* < 0.001). The number of TO interactions was higher in the Stress group than in the Sham group at R1 (*post-hoc* Bonferroni test *p* < 0.01) but not at R2. Finally, the cumulative duration of TO interaction was similar in the Stress and Sham groups at both R1 and R2.

#### 3.1.2. Behavioral evaluation

**Figure 3:**
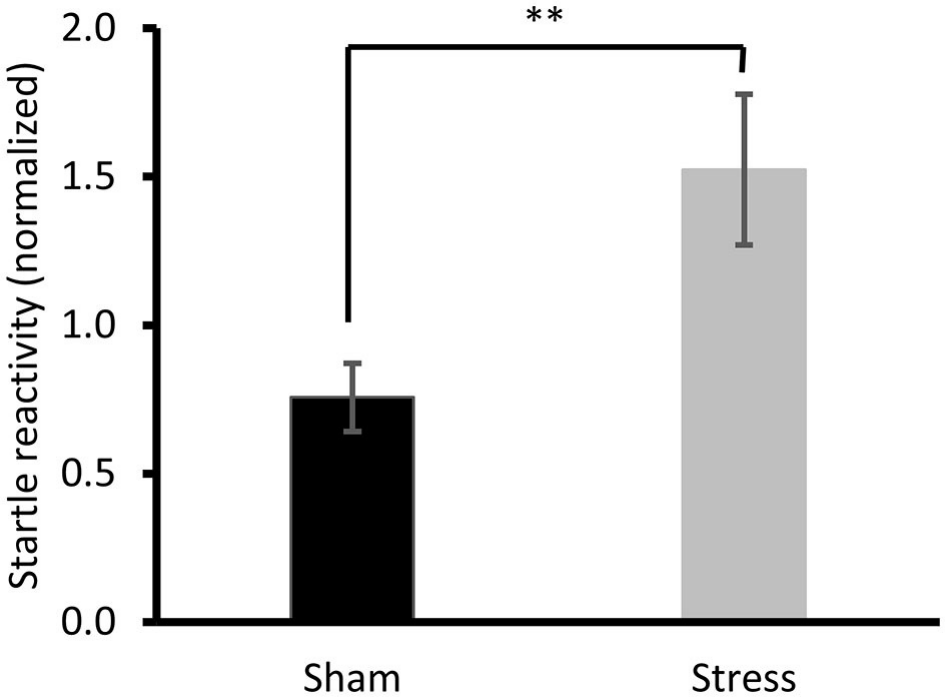
Startle response. *Post-hoc* Bonferroni test **p < 0.01. Results are expressed as mean ± SEM.

The open-field analysis found no inter-group differences [factorial ANOVA: *F*_(1, 44)_ = 0.57, ns, data not shown]. Similarly, no difference was observed in the SPT time course [repeated measures ANOVA: Group × Time effect *F*_(1, 37)_ = 0.025, ns, data not shown]. However, the startle response was higher in the Stress than in the Sham group [factorial ANOVA: *F*_(1, 44)_ = 7.35, *p* < 0.01; Figure 3].

#### 3.1.3. Biological analysis

At sacrifice, serum corticosterone levels were slightly lower in the Stress than in the Sham group [factorial ANOVA: *F*_(1, 43)_ = 2.92, *p* = 0.09, data not shown], while the opposite was observed for sBDNF [factorial ANOVA: *F*_(1, 43)_ = 3.88, *p* = 0.055, data not shown].

#### 3.1.4. Telemetric variables

**Figure 4:**
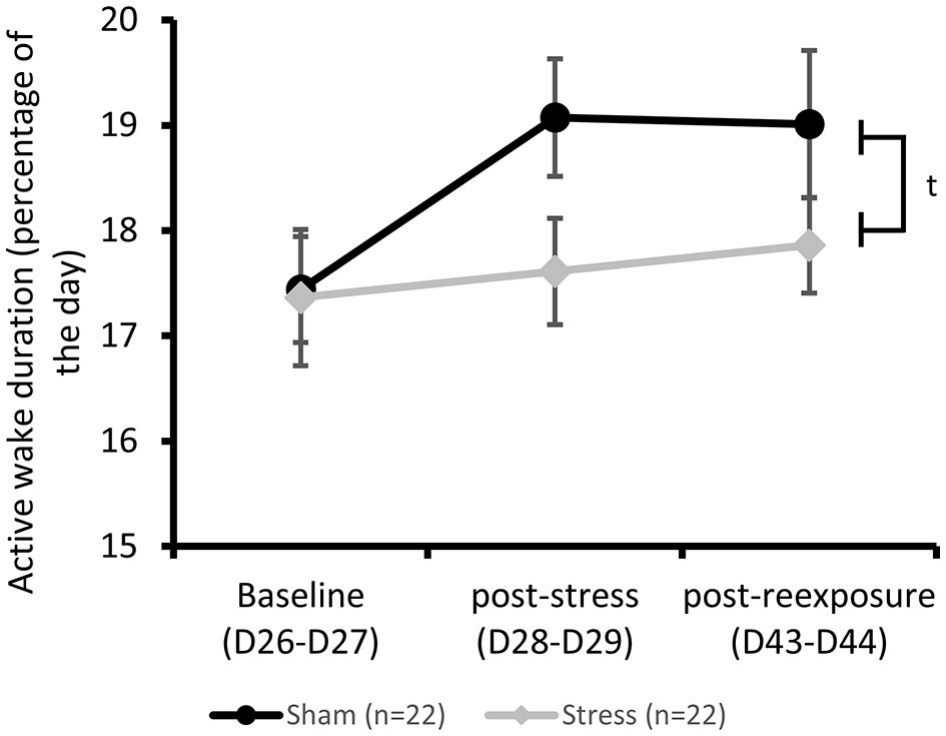
Active wake characterization: Active wake duration measured by telemetry. ANOVA for repeated measures, group effect: ^*t*^*p* < 0.10. Results are expressed as mean ± SEM.

No difference between the Stress and Sham groups was observed for active wake duration at the three experimental times, despite the overall longer waking duration in the Stress group compared to the Sham group [repeated measures ANOVA: Group effect *F*_(1, 43)_ = 3.22, *p* = 0.08; Time effect *F*_(2, 84)_ = 2.21, *p* = 0.12; Group × Time effect *F*_(2, 84)_ = 0.89 *p* = 0.41; Figure 4].

### 3.2. Stress subgroup behavioral clustering

Since the optimal number of Gaussian-repartitioned clusters was identified as three, the Stress group was divided into three Stress subgroups: (i) **C1** (Fear, 39% of animals, *n* = 9) was characterized by a high percentage of freezing behavior in the absence of unconditioned stressors; (ii) **C2** (Avoidance, 26%, *n* = 6) was characterized by a low percentage of freezing behavior and a low number and duration of TO interactions; and (iii) **C3** (Neotic, 35%, *n* = 8) was characterized by a low percentage of freezing behavior and a high number and duration of TO interactions. We named this last cluster “Neotic” because, at R2, the behavior of these rats was characterized by curiosity (neophilia) rather than avoidance (neophobia) or fear (Hughes, 2007).

**Figure 5:**
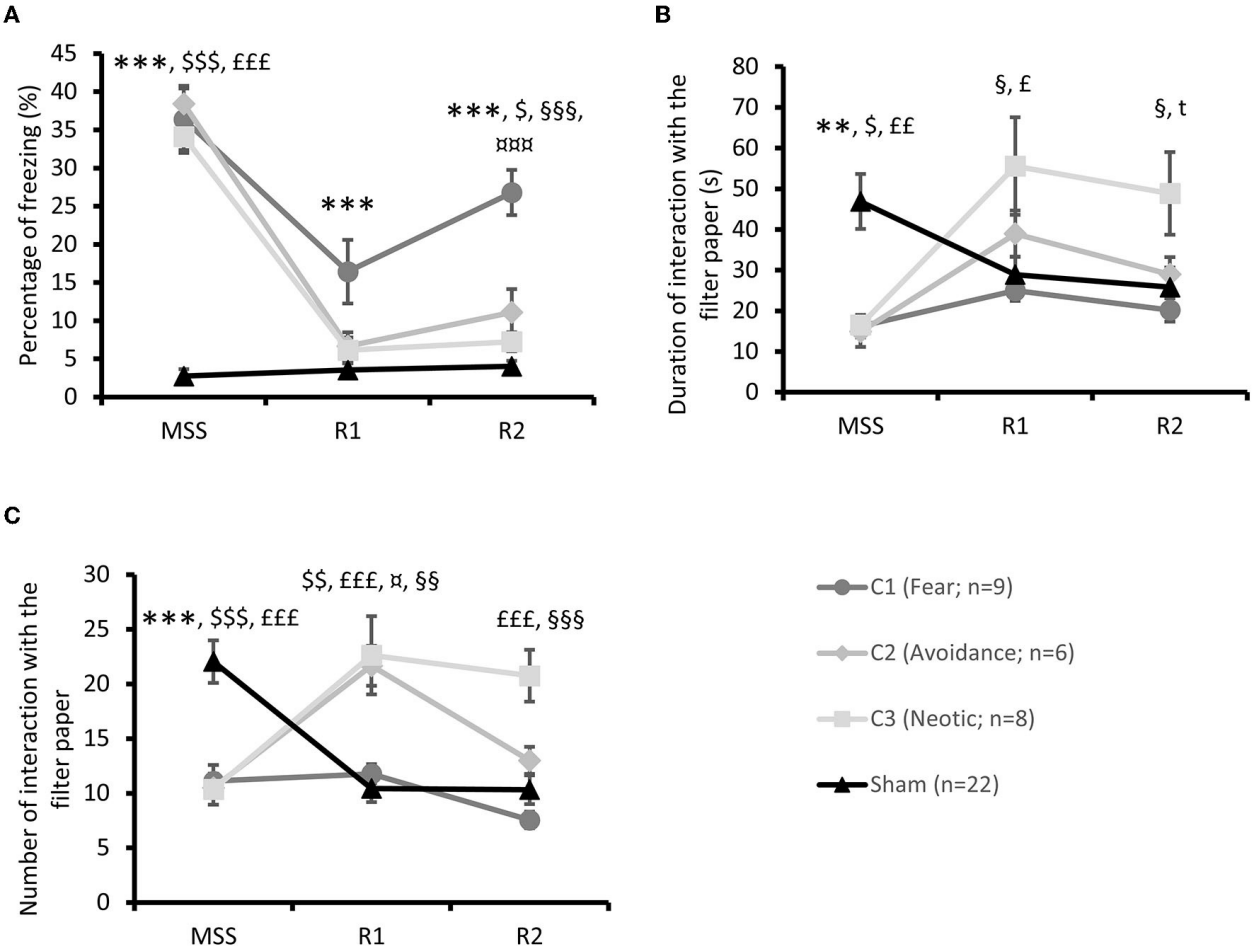
Stress group clustering. **(A)** Percentage of freezing during MSS, and R1 and R2 re-exposures. *Post-hoc* tests ***p < 0.001 between the C1 subgroup (Fear) and the Sham group, ^$^*p* < 0.05 between the C2 subgroup and the Sham group, ^$$$^*p* < 0.001 between the C2 subgroup (Avoidance) and the Sham group,^£££^*p* < 0.001 between the C3 subgroup (Neotic) and Sham group, ^§§§^*p* < 0.001 between the C1 and C3 subgroups, ^¤¤¤^*p* < 0.001 between the C1 and C2 subgroups. **(B)** Duration in seconds of interaction with TO during MSS, and R1 and R2 re-exposures. *Post-hoc* tests **p < 0.01 between the C1 subgroup and the Sham group, ^$^*p* < 0.05 between the C2 subgroup (Avoidance) and Sham group,^£^*p* < 0.05 between the C3 subgroup (Neotic) and Sham group,^££^*p* < 0.01 between the C3 subgroup (Neotic) and Sham group, ^§^*p* < 0.05 between the C1 and C3 subgroups, ^*t*^*p* < 0.10 between the C3 subgroup and the Sham group. **(C)** Number of interactions with TO during MSS, and R1 and R2 re-exposures. *Post-hoc* tests ***p < 0.001 between the C1 subgroup and the Sham group, ^$$^*p* < 0.01 between the C2 subgroup and the Sham group, ^$$$^*p* < 0.001 between the C2 subgroup and the Sham group,^£££^*p* < 0.001 between the C3 subgroup and the Sham group, ^¤^*p* < 0.05 between the C1 and C2 subgroups, ^§§^*p* < 0.01 between the C1 and C3 subgroups, ^§§§^*p* < 0.001 between the C1 and C3 subgroups. Results are expressed as mean ± SEM.

The three Stress subgroups only differed in their freezing percentage [Figure 5A; repeated measures ANOVA: Group × Time effect *F*_(4, 40)_ = 4.94; *p* < 0.001, partial Eta-squared = 0.33] at R2 with higher percentages in the C1 than the C2 subgroup (*post-hoc* Bonferroni test *p* < 0.001) or C3 subgroups (*p* < 0.001).

Compared to the Sham group, the three Stress subgroups differed [repeated measures ANOVA: *F*_(6, 82)_ = 33.61; *p* < 0.001] at MSS (*post-hoc* Dunnett test *p* < 0.001 for each); only C1 differed from the Sham group at R1 (*p* < 0.001) and at R2 (*p* < 0.001), and the C2 (*p* < 0.05) subgroup for the freezing percentage.

Furthermore, the duration of TO interactions only differed between the three Stress subgroups [Figure 5B; repeated measures ANOVA: Group × Time effect *F*_(4, 40)_ = 2.40; *p* = 0.07, partial Eta-squared = 0.19] at R1 (C1 × C3, *p* < 0.05) and R2 (C1 × C3, *p* < 0.05).

Compared to the Sham group on the duration of TO interactions, the three Stress subgroups differed [repeated measures ANOVA: *F*_(6, 82)_ = 5.62; *p* < 0.001] at MSS (*post-hoc* Dunnett test: C1 × Sham *p* < 0.01; C2 × Sham *p* < 0.05; C3 × Sham *p* < 0.01). At R1 (*p* < 0.05) and R2 (*p* = 0.08), the C3 subgroup alone differed from Sham.

Finally, the number of TO interactions differed between the three Stress subgroups [Figure 5C; repeated measures ANOVA: Group × Time effect *F*_(4, 40)_ = 7.66; *p* < 0.001, partial Eta-squared = 0.43] at R1 (C1 × C2 *p* < 0.05 and C1 × C3 *p* < 0.01) and at R2 (C1 × C3 *p* < 0.001).

Compared to the Sham group, the number of TO interactions was lower in all three Stress subgroups [repeated measures ANOVA: *F*_(6, 82)_ = 14.90; *p* < 0.001] at MSS (*post-hoc* Dunnett test *p* < 0.001 for each). At R1, both the C2 (*p* < 0.01) and C3 (*p* < 0.001) subgroups interacted with the TO more than the Sham group. At R2, the C3 subgroup interacted more with the TO than the Sham group (*p* < 0.001).

### 3.3. Behavior, biological, and ECoG subgroup characterization

#### 3.3.1. Behavioral characterization

The three Stress subgroups were analyzed in greater detail using behavioral tests and telemetric assessments. At baseline, there was no difference in time spent in the center of the open field among the subgroups [factorial ANOVA: *F*_(2, 22)_ = 0.08; *p* = 0.92, data not shown].

**Figure 6:**
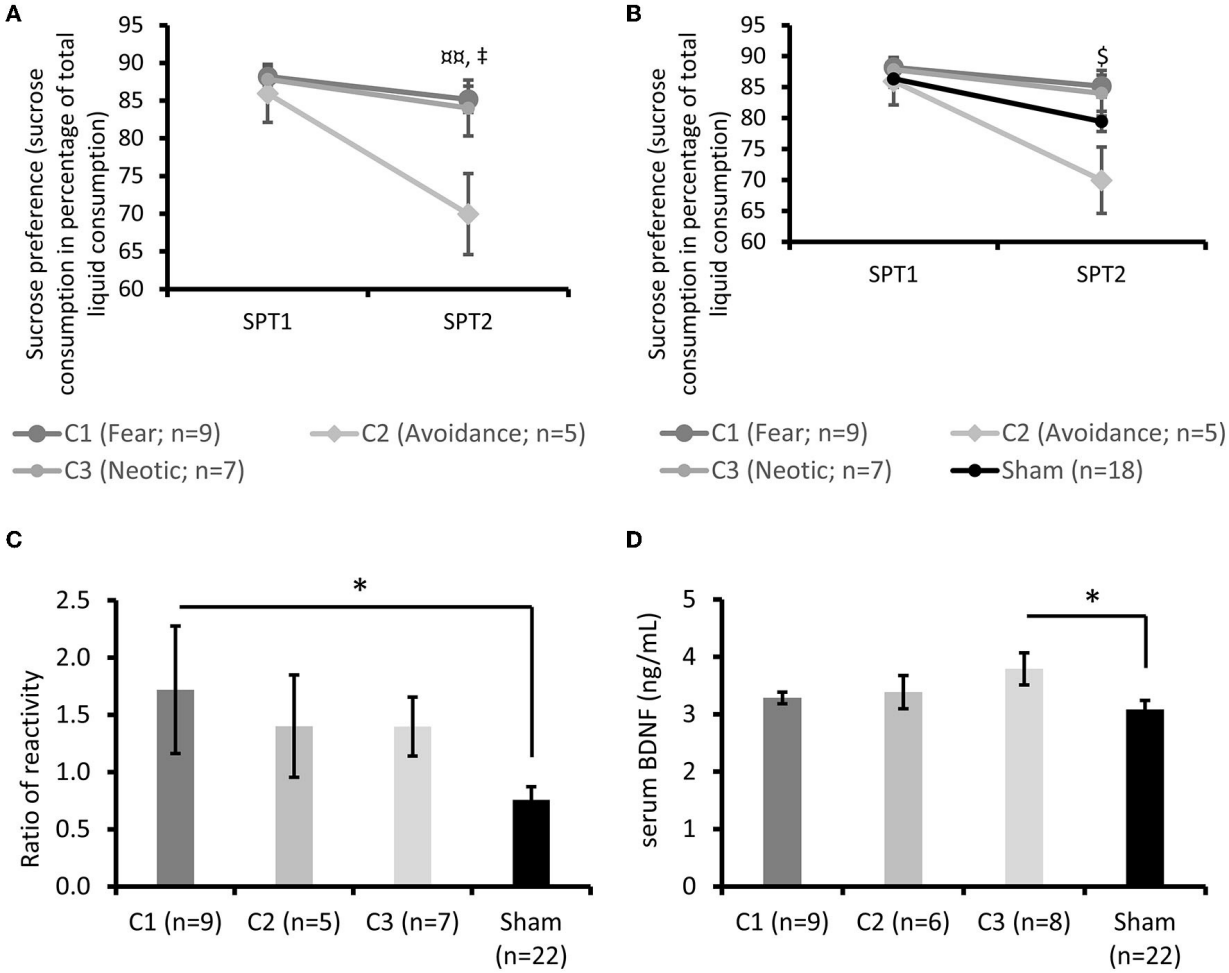
Behavioral and biological subgroup characterization. **(A)** Sucrose preference tests before and after stress exposure. *Post-hoc* test ^¤¤^*p* < 0.01 between the C1 (Fear) and C2 (Avoidance) subgroups,^‡^*p* < 0.05 between the C2 and C3 (Neotic) subgroups. **(B)** Sucrose preference tests before and after stress exposure. ^$^*p* < 0.05 between the C2 subgroup and the Sham group. **(C)** Startle reactivity compared to the Sham group. *Post-hoc* test *p < 0.05. **(D)** sBDNF at death compared to the Sham group. *Post-hoc* test *p < 0.05. Results are expressed as mean ± SEM.

During SPT2, a lower sucrose intake was observed in the C2 subgroup compared to the C1 (*post-hoc* Bonferroni test *p* < 0.01) and C3 (*p* < 0.05) subgroups [repeated measures ANOVA: Group × Time effect *F*_(2, 18)_ = 3.47 *p* < 0.05; Figure 6A]. No difference was observed at SPT1. Furthermore, the sugar intake of the C2 subgroup at SPT2 was lower than that of the Sham group [repeated measures ANOVA: Group × Time effect; *F*_(3,35)_ = 2.23, *p* = 0.10, Dunett test *p* < 0.05; Figure 6B].

No differences were observed between the three subgroups for the startle response [factorial ANOVA: *F*_(2, 22)_ = 0.17; *p* = 0.84; data not shown]. However, startle response was higher in the C1 subgroup compared to the Sham group [factorial ANOVA: *F*_(3, 41)_ = 2.57, *p* = 0.067, Dunnett test *p* < 0.05; Figure 6C].

#### 3.3.2. Biological characterization

At sacrifice, serum corticosterone was similar within the three Stress subgroups [factorial ANOVA: *F*_(2, 20)_ = 0.27, *p* = 0.77, data not shown] and between the Stress subgroups and the Sham group (data not shown). sBDNF concentrations were similar among the Stress subgroups [factorial ANOVA: *F*_(2, 20)_ = 1.54, *p* = 0.24, data not shown] but higher in the C3 subgroup than the Sham group [factorial ANOVA: *F*_(3, 41)_ = 2.15, *p* = 0.10, *post-hoc* Dunnett test *p* < 0.05; Figure 6D].

#### 3.3.3. Free-running psychophysiological characterization

**Figure 7:**
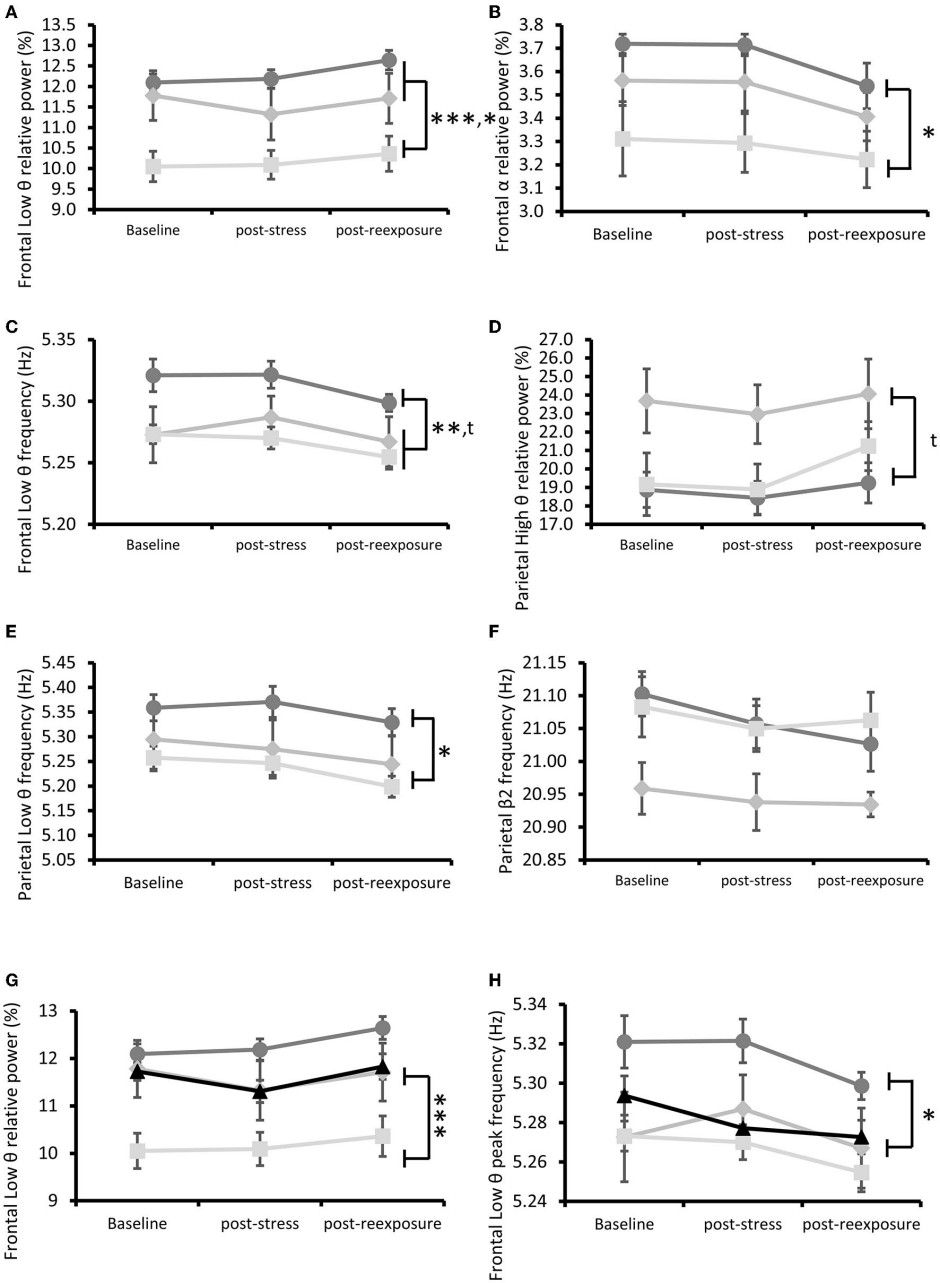
ECoG subgroup characterization throughout the experiment. **(A)** Frontal Low θ relative power. *p < 0.05 between the C3 (Neotic) and C2 (Avoidance) subgroups, ***p < 0.001 between the C1 and C3 subgroups. **(B)** Frontal α relative power. *p < 0.05 between the C1 and C3 subgroups. **(C)** Frontal Low θ frequency. **p < 0.01 between the C1 and C3 subgroups, ^*t*^*p* < 0.10 between the C1 and C2 subgroups. **(D)** Parietal High θ relative power. ^*t*^*p* < 0.10 between the C1 and C2 subgroups. **(E)** Parietal Low θ frequency. *p < 0.05 between the C1 and C3 subgroups. **(F)** Parietal β2 frequency. **(G)** Frontal Low θ relative power. Stress subgroups compared to the Sham group. *Post-hoc* test ***p < 0.001 between the C3 subgroup and the Sham group. **(H)** Frontal Low θ frequency. *Post-hoc* test *p < 0.05 between the C1 subgroup and the Sham group. Results are expressed as mean ± SEM.

Few ECoG differences were observed among the three Stress subgroups. Frontal Low θ relative power was higher in the C1 subgroup than the C2 and C3 subgroups [repeated measures ANOVA: Group effect *F*_(2, 19)_ = 11.40, *p* < 0.001, *post-hoc* Bonferroni test *p* < 0.10 and *p* < 0.01, respectively; without the Group × Time effect *F*_(4, 38)_ = 0.87, *p* = 0.49; Figure 7A]. The C1 subgroup was also characterized by a higher frontal α relative power than the C3 subgroup [repeated measures ANOVA: Group effect *F*_(2, 19)_ = 4.80, *p* < 0.05, *post-hoc* Bonferroni test *p* < 0.05; without the Group × Time effect *F*_(4, 38)_ = 0.23, *p* = 0.92; Figure 7B]. Frontal Low θ frequency was also lower in the C1 than in the C3 subgroup and tended to be lower than the C2 subgroup [repeated measures ANOVA: Group effect *F*_(2, 19)_ = 6.80, *p* < 0.01, *post-hoc* Bonferroni test *p* < 0.01 and *p* < 0.10, respectively, without the Group × Time effect *F*_(4, 38)_ = 0.38, *p* = 0.82; Figure 7C]. Parietal High θ relative power tended to be lower in the C1 subgroup than the C3 subgroup [repeated measures ANOVA: Group effect *F*_(2, 19)_ = 2.97, *p* = 0.07, *post-hoc* Bonferroni test *p* < 0.10, without the Group × Time effect *F*_(4, 38)_ = 2.02 *p* = 0.11; Figure 7D]. Parietal Low θ frequency was also higher in the C1 than in the C3 subgroup [repeated measures ANOVA: Group effect, *F*_(2, 19)_ = 3.98, *p* < 0.05, *post-hoc* Bonferroni test *p* < 0.05, without the Group × Time effect *F*_(4, 38)_ = 0.64, *p* = 0.63; Figure 7E]. Although the parietal β2 frequency was different among the three subgroups, the *post-hoc* analysis revealed no significant *post-hoc* differences between them [repeated measures ANOVA: Group effect *F*_(2, 19)_ = 2.60, *p* < 0.10, *post-hoc* Bonferroni test *p* = 0.16; C1 × C2, *p* = 0.16; C2 × C3; without the Group × Time effect *F*_(4, 38)_ = 1.22, *p* = 0.32; Figure 7F].

The C3 subgroup was characterized by a lower frontal Low θ relative power than the Sham group [repeated measures ANOVA: Group effect, *F*_(3, 40)_ = 7.51, *p* < 0.001, *post-hoc* Dunnett test *p* < 0.001 without the Group × Time effect *F*_(6, 80)_ = 0.83, *p* = 0.55; Figure 7G], while the C1 subgroup was characterized by a higher frontal Low θ frequency than the Sham group [repeated measures ANOVA: Group effect, *F*_(3, 40)_ = 3.30, *p* < 0.05, *post-hoc* Dunnett test *p* < 0.05; without the Group × Time effect *F*_(6, 80)_ = 0.84, *p* = 0.54; Figure 7H].

All relative power and frequency ECoG results are shown in Supplementary Figures 2, 3.

### 3.4. Baseline variable predictivity of subgroups

#### 3.4.1. Variable selection

**Figure 8:**
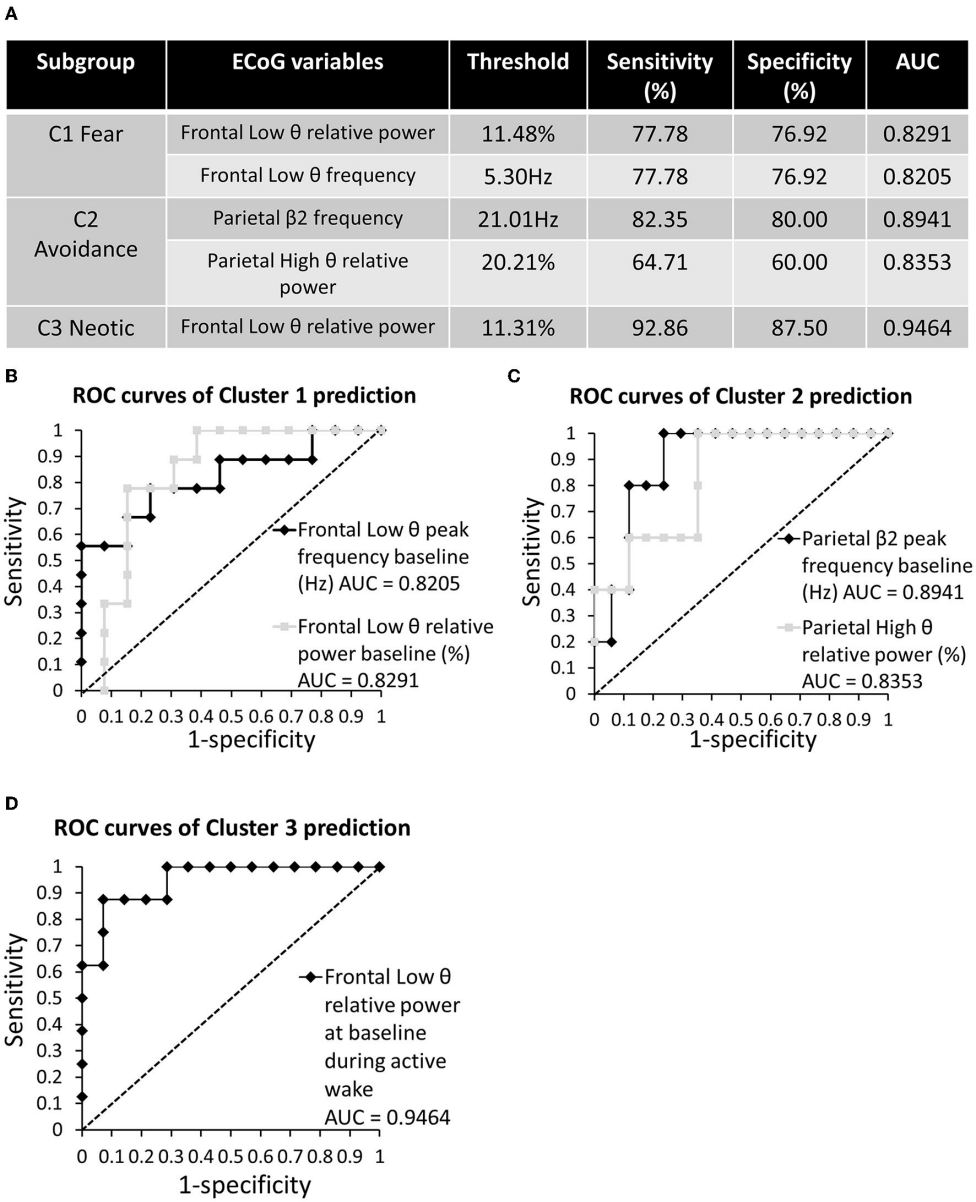
ROC curves for cluster membership predicted using baseline EcoG. **(A)** Summary of ECoG variables predicting cluster membership with AUC > 0.8. **(B)** ROC curves for C1 (Fear) predictivity. **(C)** ROC curves for C2 (Avoidance) predictivity. **(D)** ROC curves for C3 (Neotic) predictivity.

All 28 ECoG calculated variables (listed in Supplementary Figures 2, 3) were tested for their ability to predict cluster membership at baseline (D26–D27). Predictors with an AUC of > 0.8 are shown in Figure 8A.

The analysis highlighted that Cl membership could be predicted by frontal Low θ relative power and frequency peaks (threshold = 11.48%, AUC = 0.8291, sensitivity = 77.78%, specificity = 76.92%; threshold = 5.30 Hz, AUC = 0.8205, sensitivity = 77.78%, specificity = 76.92%; respectively; Figure 8B). Meanwhile, C2 membership could be predicted by the parietal β2 peak and High θ relative power (threshold = 21.01 Hz, AUC = 0.8941, sensitivity = 82.35%, specificity = 80%; threshold = 20.21%, AUC = 0.8353, sensitivity = 64.71%, specificity = 60.00%, respectively; Figure 8C). Finally, C3 membership could be predicted by frontal Low θ relative power (threshold = 11.31%, AUC = 0.9464, sensitivity = 92.86%, specificity = 87.50%; Figure 8D).

#### 3.4.2. Heuristic value of variables

Baseline frontal Low θ peak frequency was correlated with R2 freezing behavior (*r* = 0.45, *p* < 0.05) and the number of TO interactions (*r* = −0.44, *p* < 0.05). Baseline frontal Low θ relative power was correlated with R2 freezing behavior (*r* = 0.52, *p* < 0.05), and the duration (*r* = −0.51, *p* < 0.05) and number (*r* = −0.62, *p* < 0.01) of TO interactions. No correlation was observed between behavior and baseline parietal β2 frequency, or parietal High θ relative power.

## 4. Discussion

The first main result of our study is that one exposure to multisensorial stress (MSS) was followed by three different outcomes, evidenced both by behaviors observed during re-exposure and by changes in free-running ECoG functioning. The first outcome (C1, Fear) mimicked PTSD, with a significant fear response (freezing, avoidance), together with trauma memory (freezing, avoidance). The second outcome (C2, Avoidance) mimicked partial PTSD, with only trauma memory (avoidance), while the third outcome (C3, Neotic) resembled post-traumatic recovery, with no specific alteration. The second main result was the retrospective determination at baseline of the telemetric correlates of the three Stress subgroups. This result opened the way to the prospective determination of vulnerability/resilience subgroups.

### 4.1. Stress-induced pathologies

#### 4.1.1. The MSS model

Our MSS model was developed to overcome the problem of differences in stressor aggressiveness during stress exposure while maintaining the realistic and ecological aspect of the stressor. The selected stressor is a virtual exposure to a predator attack using realistic, easily reproducible cues. The scene included predator odor, blood odor, and conspecific distress vocalizations that mimicked a predator attack and were likely to engender fear (Rosen et al., 2015; Verbitsky et al., 2020). The degree of influence of each of these stimuli has not been evaluated in this experiment but was previously reported. TMT odor exposure is known to induce fear but not fear conditioning in rats (Day et al., 2004; Fendt and Endres, 2008; Horii et al., 2010), while TED mimics the odor of conspecific blood in both rodents (Stevens and Saplikoski, 1973; Lahger and Laska, 2018) and humans (Kline and Rausch, 1985; Arshamian et al., 2017). This olfactive background was reinforced by 22 kHz conspecific rat distress vocalizations that may induce fear (Fendt et al., 2018; Brudzynski, 2019), behavioral inhibition (Fendt et al., 2018), and higher startle reactivity (Inagaki and Ushida, 2017). TMT exposure was achieved by soaking the liquid in a filter paper, which became a “traumatic object” (TO). This object could become a contextual reminder of past trauma, potentially triggering an emotional memory. In brief, contextual reminders of a traumatic memory were included in a context of fear, induced by predator cues, potentially triggering defensive mechanisms in rodents (Blanchard and Blanchard, 1989). This animal model may correspond to witnessing an attack, as the animal was not itself attacked. Such a situation is known to induce PTSD in humans (Yehuda et al., 2015).

All stimuli were chosen to activate the brain areas involved in defensive mechanisms, in other words, the response to exposure to unconditioned stimuli (Blanchard and Blanchard, 1989; Blanchard et al., 1990). Although cat odor activates more stress-related brain areas than TMT in rats (Staples et al., 2008), it mostly activates the dorsal pre-mammillary nucleus, the locus coeruleus, and the VPAG, and, to a lesser extent, the amygdala and the hippocampus (Staples et al., 2005). Similarly, 22 kHz calls increase cFos mRNA expression in the inferior colliculus, the auditory cortex, the periaqueductal gray matter, the basolateral amygdala, and the hippocampus (Ouda et al., 2016). The aim of the study was to realize an ethologically relevant stress exposition to mimic human pathology. Therefore, a realistic exposition that included various stimuli was necessary.

#### 4.1.2. Behavior during MSS exposure

Our hypotheses underlying the design of the MSS model were validated by the behavior of rats during exposure. All exposed rats exhibited fear behaviors, notably freezing and TO avoidance (measured as the number and cumulative duration of TO interactions).

#### 4.1.3. Long-term sequelae

The main long-term sequela was emotional memorization. It was studied at R1 and R2 during contextual re-exposure using two dimensions: fear memory, evidenced by freezing; and contextual memory, evidenced by TO interactions. Various levels of these dimensions were observed in all rats: some rats exhibited both, only one, or none. This variability is unsurprising and has been widely reported (Ritov et al., 2016; Blount et al., 2023), although the distribution of the different behaviors varies according to the type of stressor, the variables used, the experimental design, the strain of animals, etc. These results are in line with human observations showing a wide range of pathologies corresponding to our different groups, from resilient to partial or complete PTSD (Mota et al., 2016).

### 4.2. Stress-induced clustering

Variability in stress-induced sequelae suggested that clustering would be a useful strategy to identify homogenous groups of rats according to a given symptomatology and, consequently, a potential pathology. For instance, TMT induces anxiety (enhanced startle and anxiety behaviors in the elevated plus maze) to a significant (22%), intermediate (56%), and minimal (22%) extent in animals (Schwendt et al., 2018). An underwater trauma-based model (also using Sprague-Dawley rats) led to the identification of three phenotypes that resemble our clusters (38% fear, 15% fear-anhedonic, and 47% unaffected; Ritov et al., 2016; Ritov and Richter-Levin, 2017). In our approach, we studied emotional regulation as fear, and emotional memory as TO avoidance, which is why we used behavioral indexes (freezing percentage, and the number and duration of TO interactions at R2) to isolate our three subgroups. Interestingly, the C1 subgroup was characterized by both fear (freezing) and avoidance (few, short-duration TO interactions), the C2 subgroup was only characterized by avoidance, and C3 was characterized by the absence of fear and emotional memory.

MSS exposure did not modify sucrose intake in the C1 or C3 subgroups but reduced it in C2. Such a result is unsurprising in C3, as these animals recovered completely, and is consistent with their increased sBDNF level. For C1, earlier work has identified a fear phenotype without comorbid anhedonia (Ritov et al., 2016; Ritov and Richter-Levin, 2017). Anhedonia, associated with avoidance, as observed in the C2 subgroup, suggests that the habenula may have been targeted, since this brain area is involved in flexible (Hones and Mizumori, 2022) and motivated (Hikosaka et al., 2008; Hikosaka, 2010) behaviors, fear (Durieux et al., 2020), context/ emotion interactions during memory tasks (Baker et al., 2022), and avoidance behavior (Stamatakis et al., 2013). In these cases, it is possible that there is an alteration in the dopaminergic drive involving the habenula (Friedman et al., 2011). While C1 was not characterized by anhedonia, it was the only cluster that was characterized by higher reactivity than the Sham group, suggesting an increased amygdala reactivity (Grillon, 2002; Cano et al., 2021).

### 4.3. The existence of an endophenotype

#### 4.3.1. Variables of interest

As our evidence suggests that exposure to a reproducible stressor can induce various pathologies, the question that arises is whether these differences are associated with different brain functions. Resting-state functional connectivity has been found to predict fear response (Dopfel et al., 2019), and the parietal frequency of the main β2 peak and High θ relative power has been found to predict vulnerability to depression in a social defeat model (Claverie et al., 2016). Therefore, we looked for differences in baseline ECoG that would predict the membership of one of the three stressed subgroups. Then, we checked the power of each ECoG variable to predict subgroup membership.

#### 4.3.2. Cluster correlates

A ROC analysis goes further than observations of differences between clusters and can be used to assess a variable's progressive ability to predict cluster membership. Baseline frontal Low θ relative power and frequency peaks were able to discriminate between the membership of C1 (high values) and C3 (low values). Low frontal Low θ relative power, together with low frontal α, suggests that frontal cortex activity is modest in the C3 subgroup but high in C1. Furthermore, frontal Low θ relative power could discriminate between C3 and C1 and C2, whereas frontal Low θ peak frequency could discriminate between C1 and C2 and C3. It is reasonable to believe that enhanced relative power is related to avoidance, whether efficient (C2) or not (C1), whereas frontal Low θ peak frequency is associated with fear regulation, whether efficient (C2 and C3) or not (C1). Moreover, increased θ activity, with frontal, temporal, and parietal synchrony, has been reported in PTSD patients compared to healthy controls (Dunkley et al., 2015). Increased θ activity is a marker of atypical network interactions in PTSD patients, and it appears that the θ band could play a critical role in the temporal coordination of information required for effective cognitive flexibility (Dunkley et al., 2015).

Our experiment found that higher values for Low θ power were not specific to the PTSD phenotype but were markers of non-resilient phenotypes (C1 and C2), and, conversely, lower values were a hallmark of resilience. Our result is in line with earlier observations of increased frontal θ power in MDD (Arns et al., 2015; Zhang et al., 2022), demonstrating that this biomarker is not specific to a pathology. However, our study goes further and shows that ECoG differences can be observed before stress exposure and can be used to predict phenotypes. Their stability over time suggests that they could be a marker of a vulnerability endophenotype. Altogether, our findings suggest that high frontal cortex Low θ power is associated with poor recovery from MSS exposure and could be a hallmark of abnormal brain synchrony.

The parietal frequency of the main β2 peak and parietal High θ relative power were identified as predictors of C2 membership. This is unsurprising as the same variables have been associated with vulnerability to depression (Claverie et al., 2016). However, the parietal ECoG assessment raises the question of whether there is interference between locomotion and θ as parietal High θ relative power is related to the C2 subgroup.

Overall, the variables assessed at the baseline: (i) predict membership of a subgroup; (ii) distinguish between subgroups; and (iii) remain stable throughout the investigation. Therefore, they appeared to be able to detect the membership of a particular subgroup. A few variables are of particular interest: frontal Low θ relative power and the parietal frequency of the main β2 peak performed best in our ROC analysis and at detecting differences between subgroups.

## 5. Limitations

We are unable to conclude whether cluster membership is independent of stress perceptions. Nevertheless, the lack of a difference between the Stress subgroups concerning freezing and avoidance during stress exposure confirms the intensity of the situation and the perception of a threat. Future work would include measures such as heart rate variability to evaluate sympathetic activation and parasympathetic inhibition during stress exposure to confirm the level of the physiological reactions.

## 6. Conclusion

Our multisensorial stress model was able to induce a significant stress response, and the outcomes mimicked fear conditioning, avoidance, and full recovery. These outcomes can be linked to baseline ECoG biomarkers that remained stable throughout the investigation and reliably predicted behavioral subgroup membership. The following step would be the assessment of the biological and molecular changes that characterized each cluster before stress exposure. It would be particularly helpful to be able to use these characteristics to identify the therapeutic targets of PTSD vulnerability. Moreover, the selected ECoG biomarkers were characterized by a stable time course, which underlines their reliability. These ECoG biomarkers open the way to personalized preventive medicine. Prevention is important for individuals repeatedly exposed to occupational attacks (soldiers, firefighters...).

## Data availability statement

The original contributions presented in the study are included in the article/Supplementary material, further inquiries can be directed to the corresponding author.

## Ethics statement

The animal study was reviewed and approved by IRBA Institutional Ethic Committee (Authorization No: 24.2017).

## Author contributions

LD, BP, ME, MT, NT, FC, and DCl contributed to conception and design of the study. LD, BP, ME, CA, DCo, FB, CLe, FF, CLi, PC, AD, AS, OF, NT, and DCl contributed to the acquisition. LD, FC, and DCl performed the statistical analysis and wrote the manuscript. All authors contributed to manuscript revision, read, and approved the submitted version.
