# Supplementary material for: Steady electrocorticogram characteristics predict specific stress-induced behavioral phenotypes
Source: Front Neurosci. 2023 Apr 11;17:1047848. doi: 10.3389/fnins.2023.1047848 (PMC10126346; doi:10.3389/fnins.2023.1047848)
Supplement: Supplementary file 1 [file Data_Sheet_1.PDF]

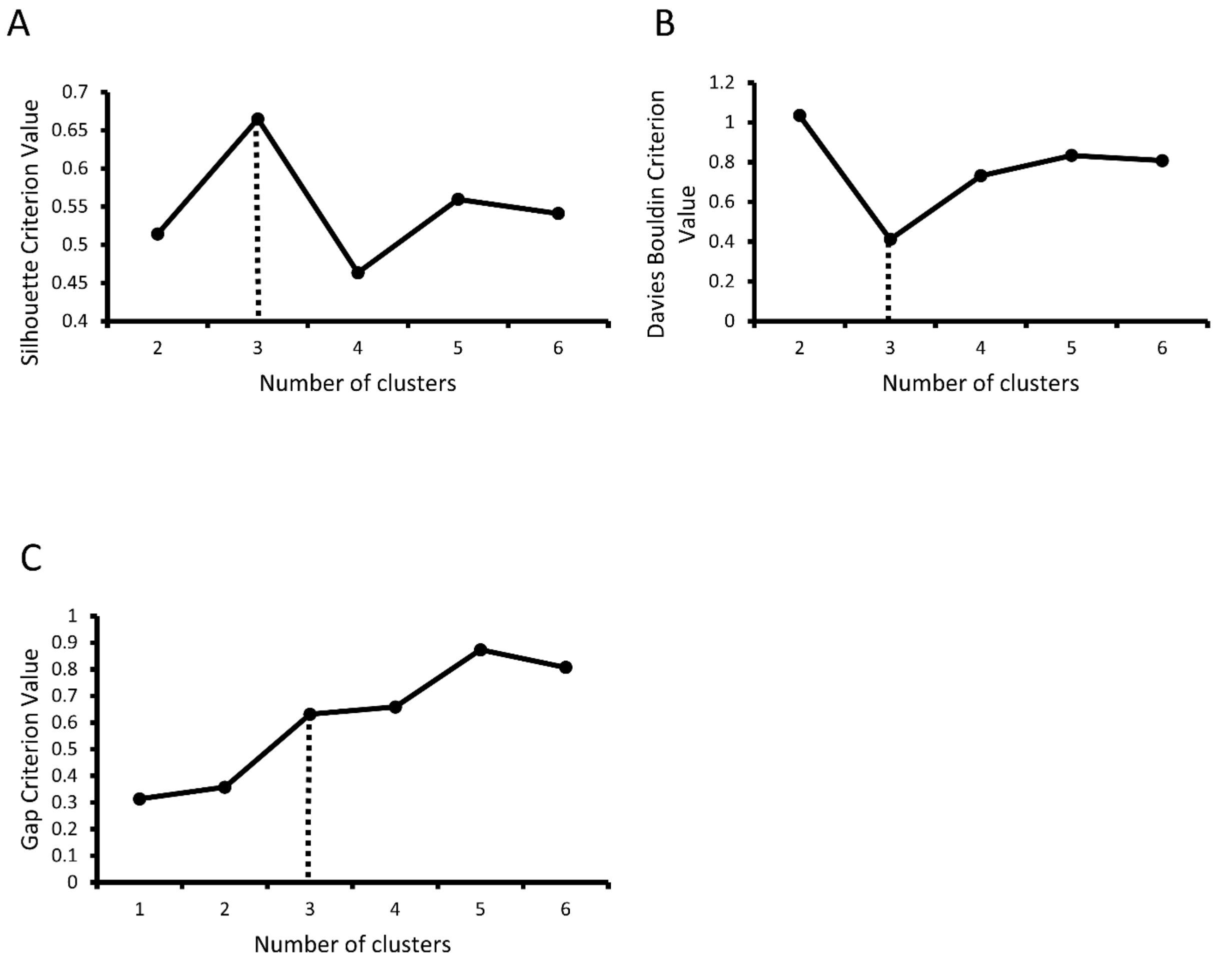

Supplementary Figure 1: Optimal number of clusters in stressed animals for a Gaussian Mixture Model. A. Silhouette Criterion Values in function of number of clusters. **B**. Davies Bouldin Criterion Values in function of number of clusters. **C**. Gap Criterion Values in function of number of clusters.
