# Supplementary material for: Steady electrocorticogram characteristics predict specific stress-induced behavioral phenotypes
Source: Front Neurosci. 2023 Apr 11;17:1047848. doi: 10.3389/fnins.2023.1047848 (PMC10126346; doi:10.3389/fnins.2023.1047848)
Supplement: Supplementary file 2 [file Data_Sheet_2.PDF]

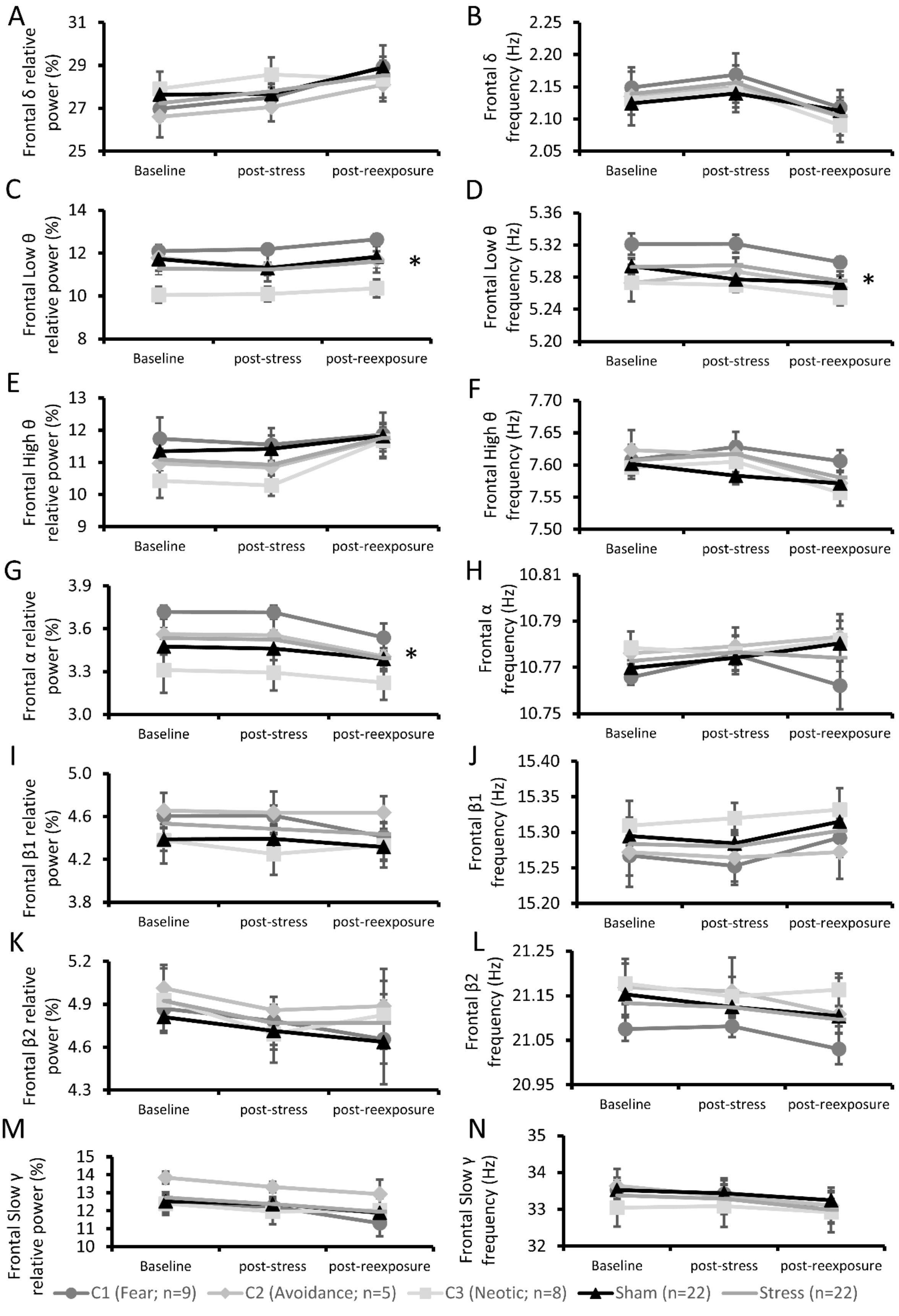
A
Frontal δ relative power (%)
B
Frontal δ frequency (Hz)
C
Frontal Low θ relative power (%)
D
Frontal Low θ frequency (Hz)
E
Frontal High θ relative power (%)
F
Frontal High θ frequency (Hz)
G
Frontal α relative power (%)
H
Frontal α frequency (Hz)
I
Frontal β1 relative power (%)
J
Frontal β1 frequency (Hz)
K
Frontal β2 relative power (%)
L
Frontal β2 frequency (Hz)
M
Frontal Slow γ relative power (%)
N
Frontal Slow γ frequency (Hz)
Baseline
post-stress
post-reexposure
*
C1 (Fear; n=9)
C2 (Avoidance; n=5)
C3 (Neotic; n=8)
Sham (n=22)
Stress (n=22)

Supplementary Figure 2: Frontal ECoG subgroup characterization. A. Frontal δ relative power during the experiment. **B**. Frontal δ main frequency during the experiment. **C**. Frontal Low θ relative power during the experiment. *: observed significant results (cf Figure 7.A and 7.G: C3 neotic *vs* C1 fear: $p < 0.001$, C3 neotic *vs* C2 avoidance: $p < 0.05$, C3 neotic vs Sham group). **D**. Frontal Low θ main frequency during the experiment. *: observed significant results (Figure 7.C: C3 neotic *vs* C1 fear: $p < 0.01$, C1 fear *vs* C2 avoidance: $p < 0.10$, C1 fear *vs* Sham group). **E**. Frontal High θ relative power during the experiment. **F**. Frontal High θ main frequency during the experiment. **G**. Frontal α relative power during the experiment. *: observed significant results (cf Figure 7.B: C3 neotic *vs* C1 fear: $p < 0.05$). **H**. Frontal α main frequency during the experiment. **I**. Frontal β1 relative power during the experiment. **J**. Frontal β2 main frequency during the experiment. **K**. Frontal β2 relative power during the experiment. **L**. Frontal β2 main frequency during the experiment. **M**. Frontal Slow γ relative power during the experiment. **N**. Frontal Slow γ main frequency during the experiment. Results are expressed as mean ± SEM.
