# Supplementary material for: Steady electrocorticogram characteristics predict specific stress-induced behavioral phenotypes
Source: Front Neurosci. 2023 Apr 11;17:1047848. doi: 10.3389/fnins.2023.1047848 (PMC10126346; doi:10.3389/fnins.2023.1047848)
Supplement: Supplementary file 3 [file Data_Sheet_3.PDF]

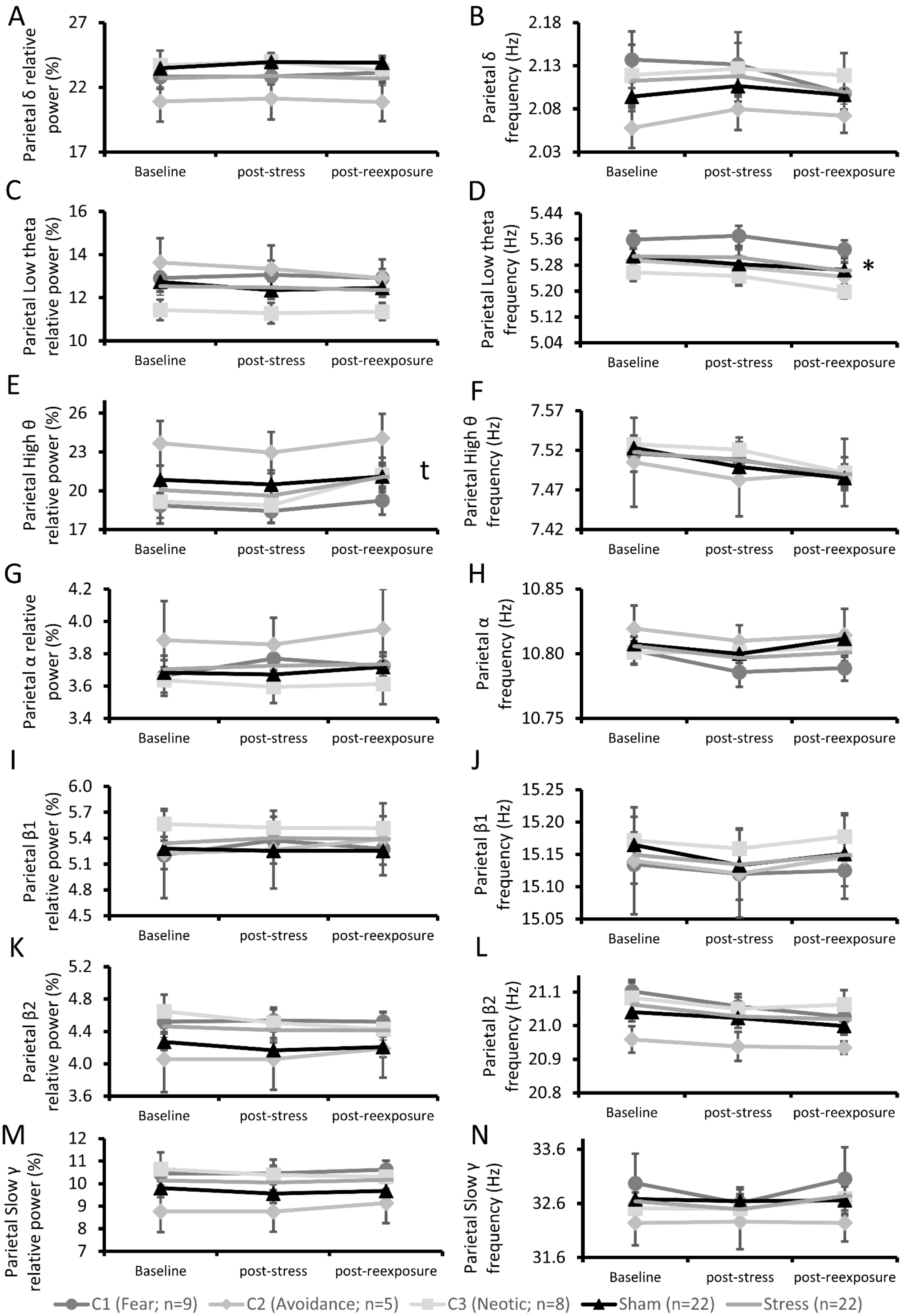
A
Parietal δ relative power (%)
27
22
17
Baseline
post-stress
post-reexposure
B
Parietal δ frequency (Hz)
2.18
2.13
2.08
2.03
Baseline
post-stress
post-reexposure
C
Parietal Low theta relative power (%)
16
14
12
10
Baseline
post-stress
post-reexposure
D
Parietal Low theta frequency (Hz)
5.44
5.36
5.28
5.20
5.12
5.04
*
Baseline
post-stress
post-reexposure
E
Parietal High θ relative power (%)
26
23
20
17
t
Baseline
post-stress
post-reexposure
F
Parietal High θ frequency (Hz)
7.57
7.52
7.47
7.42
Baseline
post-stress
post-reexposure
G
Parietal α relative power (%)
4.2
4.0
3.8
3.6
3.4
Baseline
post-stress
post-reexposure
H
Parietal α frequency (Hz)
10.85
10.80
10.75
Baseline
post-stress
post-reexposure
I
Parietal β1 relative power (%)
6.0
5.7
5.4
5.1
4.8
4.5
Baseline
post-stress
post-reexposure
J
Parietal β1 frequency (Hz)
15.25
15.20
15.15
15.10
15.05
Baseline
post-stress
post-reexposure
K
Parietal β2 relative power (%)
5.2
4.8
4.4
4.0
3.6
Baseline
post-stress
post-reexposure
L
Parietal β2 frequency (Hz)
21.1
21.0
20.9
20.8
Baseline
post-stress
post-reexposure
M
Parietal Slow γ relative power (%)
12
11
10
9
8
7
Baseline
post-stress
post-reexposure
N
Parietal Slow γ frequency (Hz)
33.6
32.6
31.6
Baseline
post-stress
post-reexposure
C1 (Fear; n=9)
C2 (Avoidance; n=5)
C3 (Neotic; n=8)
Sham (n=22)
Stress (n=22)

Supplementary Figure 3: Parietal ECoG subgroup characterization. A. Parietal δ relative power during the experiment. **B**. Parietal δ main frequency during the experiment. **C**. Parietal Low θ relative power during the experiment. **D**. Parietal Low θ main frequency during the experiment. *: observed significant results (cf Figure 7.E: C3 neotic *vs* C1 fear: $p < 0.05$). **E**. Parietal High θ relative power during the experiment. t: observed tendency (cf Figure 7.D: C2 avoidance *vs* C1 fear: $p < 0.10$). **F**. Parietal High θ main frequency during the experiment. **G**. Parietal α relative power during the experiment. **H**. Parietal α main frequency during the experiment. **I**. Parietal β1 relative power during the experiment. **J**. Parietal β2 main frequency during the experiment. **K**. Parietal β2 relative power during the experiment. **L**. Parietal β2 main frequency during the experiment. **M**. Parietal Slow γ relative power during the experiment. **N**. Parietal Slow γ main frequency during the experiment. Results are expressed as mean ± SEM.
